# S-Layer From *Lactobacillus brevis* Modulates Antigen-Presenting Cell Functions via the Mincle-Syk-Card9 Axis

**DOI:** 10.3389/fimmu.2021.602067

**Published:** 2021-03-01

**Authors:** Mariano Prado Acosta, Guillaume Goyette-Desjardins, Jörg Scheffel, Anne Dudeck, Jürgen Ruland, Bernd Lepenies

**Affiliations:** ^1^Research Center for Emerging Infections and Zoonoses, Institute for Immunology, University of Veterinary Medicine, Hannover, Germany; ^2^Dermatological Allergology, Allergie-Centrum-Charité, Department of Dermatology and Allergy, Charité – Universitätsmedizin Berlin, Corporate Member of Freie Universität Berlin, Humboldt-Universität zu Berlin, Berlin Institute of Health, Berlin, Germany; ^3^Medical Faculty, Institute for Molecular and Clinical Immunology, Otto-von-Guericke Universität Magdeburg, Magdeburg, Germany; ^4^School of Medicine, Institute of Clinical Chemistry and Pathobiochemistry, Technical University of Munich, Munich, Germany; ^5^German Cancer Consortium (DKTK), Partner Site Munich, Munich, Germany; ^6^German Center for Infection Research (DZIF), Partner Site Munich, Munich, Germany

**Keywords:** *Lactobacillus brevis*, Mincle, Syk (spleen tyrosine kinase), CARD9, S-layer, antigen presenting cell

## Abstract

C-type lectin receptors (CLRs) are pattern recognition receptors that are crucial in the innate immune response. The gastrointestinal tract contributes significantly to the maintenance of immune homeostasis; it is the shelter for billions of microorganisms including many genera of *Lactobacillus* sp. Previously, it was shown that host-CLR interactions with gut microbiota play a crucial role in this context. The Macrophage-inducible C-type lectin (Mincle) is a Syk-coupled CLR that contributes to sensing of mucosa-associated commensals. In this study, we identified Mincle as a receptor for the Surface (S)-layer of the probiotic bacteria *Lactobacillus brevis* modulating GM-CSF bone marrow-derived cells (BMDCs) functions. We found that the S-layer/Mincle interaction led to a balanced cytokine response in BMDCs by triggering the release of both pro- and anti-inflammatory cytokines. In contrast, BMDCs derived from Mincle^−/−^, CARD9^−/−^ or conditional Syk^−/−^ mice failed to maintain this balance, thus leading to an increased production of the pro-inflammatory cytokines TNF and IL-6, whereas the levels of the anti-inflammatory cytokines IL-10 and TGF-β were markedly decreased. Importantly, this was accompanied by an altered CD4^+^ T cell priming capacity of Mincle^−/−^ BMDCs resulting in an increased CD4^+^ T cell IFN-γ production upon stimulation with *L. brevis* S-layer. Our results contribute to the understanding of how commensal bacteria regulate antigen-presenting cell (APC) functions and highlight the importance of the Mincle/Syk/Card9 axis in APCs as a key factor in host-microbiota interactions.

## Introduction

The intestinal tract of mammals is colonized by a large number of microorganisms including trillions of bacteria that are collectively referred to as the gut microbiota (1). These indigenous microorganisms have co-evolved with their host in a symbiotic relationship (2). In addition to metabolic benefits for the host, these bacteria contribute to the maintenance of immune homeostasis, modulate immune responses and provide protection against pathogen colonization (3). The gut microbiome impacts host immunity by influencing the release of pro- and anti-inflammatory cytokines (4), release of metabolites (5), and by modulating functions of antigen-presenting cells, including dendritic cells (DCs) (6, 7). Recent studies show that a disturbance of the gut microbiome is associated with the progression of diseases such as inflammatory bowel disease (IBD), obesity and cancer (8–11). Moreover, in addition to local effects in the intestine, gut microbiota also influences host immune responses at extra-intestinal distant sites such as the brain, bone marrow, and lung (12–15).

There are numerous species of microorganisms present in the gut microbiota, among them *Lactobacillus* being one of the major bacterial genera found in the mammalian gut (16). *Lactobacillus* sp. belongs to the lactic acid bacteria, a broadly defined group characterized by the formation of lactic acid as the sole or main end product of carbohydrate metabolism (17, 18). In particular, *Lactobacillus* species found in the human gut have received tremendous attention due to their health-promoting properties (19). They are commonly used as probiotics, which are defined by the FAO/WHO as live microorganisms that confer a health benefit to the host, if administered in adequate amounts (20). *Lactobacillus* sp. contributes to gut immune homeostasis in many different ways; however, the mechanisms contributing to immune regulation are still incompletely understood to date.

Many species of the genus *Lactobacillus* possess Surface (S) layer proteins in their outermost envelope (21). S-layer proteins are organized into arrays of a single polypeptide non-covalently bound to the outermost envelope in the bacterial cell surface. They play a crucial role in several biological functions, such as initiation of immune responses. For instance, S-layer proteins modulate DCs and T cell functions (22) and induce the production of pro- as well as anti-inflammatory cytokines (23).

It has been previously shown that S-layer proteins interact with host C-type lectin receptors (CLRs) (24, 25). Myeloid CLRs are pattern recognition receptors (PRRs) that are crucial for innate immunity and protection from invasive pathogens by initiating innate sensing and early antimicrobial responses (26, 27). Furthermore, CLRs play an important regulatory role and maintain immune homeostasis in the gut (28). Previous work by us and others indicate that the interaction of S-layer from *Lactobacillus acidophilus* with the CLR DC-SIGN strongly inhibits viral and bacterial infections (24, 29) and induces DC effector functions (30). Moreover, the interaction between *L. acidophilus* S-layer and the murine DC-SIGN ortholog SIGNR3 exerts regulatory signals resulting in the mitigation of colitis, maintenance of gastrointestinal microbiota and gut mucosal barrier function (31).

The Macrophage-inducible C-type lectin (Mincle) is a Syk-coupled CLR. Sensing of mucosa-associated commensals by the Mincle/Syk pathway in DCs contributes to IL-6 and IL-23p19 production, thus promoting intestinal barrier function and limiting inflammation and dysregulated metabolism in the liver (3). In a recent study, it was shown that S-layer from *Lactobacillus kefiri* provokes an immunostimulatory response and adjuvant activity *in vivo* via Mincle engagement (32).

In this study, we purified S-layer from *Lactobacillus brevis*, a microorganism known to exhibit a broad spectrum of probiotic properties (33, 34). We determined the role of Mincle in S-layer sensing of this *Lactobacillus* species and analyzed how Mincle-mediated signaling affected GM-CSF bone marrow-derived cells (BMDCs) effector functions upon *L. brevis* S-layer stimulation. Interestingly, we found that Mincle significantly contributed to the production of anti-inflammatory cytokines, particularly IL-10, upon *L. brevis* stimulation in a Syk/Card9-dependent manner. Our data provide insights into the interaction of microbiota with host innate immunity and beneficial *L. brevis* contribution to immune homeostasis.

## Materials and Methods

### Isolation of S-Layer Proteins

S-layer proteins were extracted from overnight cultures of *Lactobacillus brevis (L. brevis)* ATCC 14869 bacteria grown in MRS broth at 32°C and 5% CO_2_ by using a two-step LiCl extraction; first, with 1 M LiCl to release S-layer associated proteins (SLAP), and then with 6 M LiCl (35). The protein was extensively dialyzed against distilled water overnight at 4°C and after centrifugation (10,000 × g 20 min), it was suspended in sterile H_2_O and stored at 4°C. Purity was evaluated by SDS-PAGE, which showed a single band after Coomassie blue and silver staining (Supplementary Figure 1).

### Mice

The source of the Mincle^−/−^ mice (generated by the Consortium for Functional Glycomics) and CARD9^−/−^ mice was described previously (36, 37). Inducible Syk-deficient mice (RosaCreERT2/Syk^fl/fl^) were generated by crossing B6.129P2-*Syk*^*tm*1.2*Tara*^/J mice with B6.129-*Gt(ROSA)26Sor*^*tm*1(*cre*/*ERT*2)*Tyj*^/J (both from Jackson Laboratory). Mice were bred heterozygously for the Syk allele to obtain RosaCreERT2/Syk^wt/wt^ and RosaCreERT2/Syk^fl/fl^. Mouse lines and the respective C57BL/6 wild type (WT) control mice and OT-II transgenic mice, were housed in the animal facility of the University of Veterinary Medicine Hannover under controlled temperature and humidity and specific pathogen-free conditions. Mice were sacrificed and tibia and femur from Mincle^−/−^, CARD9^−/−^, RosaCreERT2/Syk^fl/fl^, RosaCreERT2/Syk^wt/wt^ and WT mice were prepared for the isolation of bone marrow cells.

### Cell Culture

Bone marrow cells were isolated from tibia and femur of mice as previously described (38). To obtain GM-CSF bone marrow-derived cells (BMDCs), bone marrow cells were cultured in T-125 flask with IMDM complete medium supplemented with 10% FBS and 5% of GM-CSF supernatant derived from X63 cells (39). Medium was exchanged every 48 h and BMDCs were used after 8–10 days of differentiation to ascertain that ≥80% of the cell population expressed the DC marker CD11c. While the majority of cells expressed the DC marker CD11c, it should be noted that BMDC cultures are heterogeneous and also contain a substantial portion of macrophages. Thus, here and in the following, the abbreviation “BMDC” is used for “GM-CSF bone marrow-derived cells.” All cells were grown at 37°C in 5% CO_2_.

RosaCreERT2/Syk^fl/fl^ and RosaCreERT2/Syk^wt/wt^-derived BMDCs were cultured in T-125 flasks for suspension cell culture with IMDM complete medium supplemented with 10% FBS and 5% of GM-CSF supernatant. 4-hydroxytamoxifen (Sigma, #H7904) was added to the cell culture at a final concentration of 2 μM every 5 days with a complete media change leading to a conditional Syk knockdown in RosaCreERT2/Syk^fl/fl^ (Syk^−/−^), while not affecting RosaCreERT2/Syk^wt/wt^ cells (Syk^wt/wt^). Cells were treated for 14 consecutive days and were finally cultured in 4-hydroxytamoxifen-free medium for 5 days. Efficacy of Syk knockout was confirmed by PCR analysis (Supplementary Figure 2).

HEK-Blue™ mMincle cells (InvivoGen #hkb-mmcl) were cultured in T-75 flasks for adherent cell culture with DMEM medium (4.5 g/l glucose), supplemented with 10% FBS, penicillin-streptomycin (100 U/ml, 100 μg/ml), 100 μg/ml Normocin™ and 2 mM L-glutamine until a confluency of ~80% was reached.

### Stimulation of HEK-Blue™ mMincle Reporter Cells

HEK-Blue™ mMincle reporter cell stimulation was done according to the manufacturer's instructions (InvivoGen #hkb-mmcl). Briefly, after reaching confluence, the supernatant was removed and the cells were rinsed with 3 ml of PBS and afterwards resuspended in pre-warmed DMEM at 3 × 10^5^ cells/ml. 180 μl cell suspension was seeded in a 96-well plate and different concentrations of S-layer protein were added and incubated for 24 h at 37°C and 5% CO_2_. After the incubation, 180 μL of QUANTI-Blue solution (prepared by mixing 9,8 ml ddH_2_O, 0,1 ml QB reagent, 0,1 ml QB buffer) was added to a new flat-bottom 96-well plate and 20 μl of the supernatant of the previously stimulated cells was added. The mix was incubated for 3 h at 37°C and 5% CO_2_ and then the optical density (OD) was measured at 620 nm.

### Stimulation of BMDCs With S-Layer

Bone marrow cells were differentiated as described above. BMDCs were seeded at a concentration of 1 × 10^5^ cells/ml in a 96-well plate and stimulated for 24 h with S-layer at concentrations of 5 μg/ml and 10 μg/ml at 37°C and 5% CO_2_. Lipopolysaccharide (LPS) from *Escherichia coli* strain K-235 (Sigma-Aldrich) at a concentration of 1 μg/ml was added as a positive control. On the next day, supernatants were harvested and cytokine concentrations were measured by ELISA.

### Production of Mincle-hFc Fusion Protein

Murine Mincle-hFc fusion protein was produced as described previously (40). Briefly, the cDNA encoding the extracellular part of murine Mincle was amplified by polymerase chain reaction (PCR) and was then ligated into the pFuse-hIgG1-Fc2 expression vector (Invivogen #pfuse-hg1fc2) using the following primers: Mincle-FW 5′-CCATGGGGCAGAACTTACAGCCACAT-3′ and RV 5′-AGATCTGTCCAGAGGACTTATTTCTG-3′). CHO-S cells were transiently transfected with the construct using MAX reagent (InvivoGen, San Diego, California, United States). Mincle-hFc fusion protein was purified after 4 days of transfection from the cell supernatant using HiTrap protein G HP columns (GE Healthcare, Piscataway, NJ, United States). To confirm its presence and purity, the fusion protein was analyzed by SDS-PAGE and subsequent Coomassie blue staining and by Western blot using an anti-human IgG-horseradish peroxidase (HRP) antibody. As a specificity control for the binding assays, the hFc fragment was also expressed and purified in the same fashion as murine Mincle-hFc, but by using the pFuse-hIgG1-Fc2 expression vector without any cloned CLR (“empty vector”). Recombinant human Mincle-hFc (CLEC4E-Fc) was procured from R&D #8995-CL (R&D Systems, Minneapolis, MN, USA).

### ELISA-Based Binding Studies

A microplate with half-area wells (Greiner Bio-One GmbH, Frickenhausen, Germany) was coated with 50 μl of 1 μg/ml of S-layer protein overnight at RT. Non-adherent protein was washed away, and the plate was blocked with buffer containing 1% BSA (Thermo Fisher Scientific, Darmstadt, Germany) in PBS for 2 h at RT. After washing the wells, 200 ng of Mincle-hFc fusion protein in lectin-binding buffer (50 mM HEPES, 5 mM MgCl_2_, and 5 mM CaCl_2_) was added per well and incubated for 1 h at RT. Then, a 1:5,000-diluted HRP-conjugated goat anti-human IgG antibody (Dianova, Geneva, Switzerland) was added for 1 h at RT. Finally, the substrate solution [o-phenylenediamine dihydrochloride substrate tablet (Thermo Fisher Scientific, Massachusetts, United States), 24 mM citrate buffer, 0.04% H_2_O_2_, 50 mM phosphate buffer in H_2_O] was added to the samples, and the reaction was stopped with 2.0 M sulfuric acid. Data were collected using a Multiskan Go microplate spectrophotometer (Thermo Fisher Scientific, Waltham, United States) at a wavelength of 495 nm. When competition assays were performed, different concentrations of S-layer were incubated with Mincle-hFc protein and subsequently added to the wells that had been pre-coated with 50 μg/ml of Trehalose-6,6-dimycolate (TDM) (Invivogen, San Diego, United States).

### Flow Cytometry-Based Binding Assay

Flow cytometry-based binding studies were performed to detect *L. brevis*/Mincle interactions. 3 × 10^7^ CFU/ml of *L. brevis* were stained with 1 μM of the DNA-staining dye SYTO61 (Thermo Fisher Scientific) and incubated for 30 min at RT. Subsequently, samples were incubated for 1 h with 200 ng of Mincle-hFc fusion protein in lectin-binding buffer. After washing once with lectin-binding buffer, the bacterial pellet was stained with a PE-conjugated goat anti-human Fc antibody solution (Dianova, 1:200 dilution) and incubated for 25 min at 4°C. Finally, flow-cytometric analysis was performed using an Attune NxT Flow Cytometer (Thermo Fisher Scientific). Data analysis was performed using the FlowJo Software (FlowJo, Ashland, OR, USA). Additionally, *L. brevis* cells were treated with 6 M LiCl to extract the S-layer from the cell wall. As control, hFc protein was used for binding studies to exclude nonspecific binding.

### Piceatannol Treatment

Piceatannol (PC), a Syk tyrosine kinase inhibitor (ab120722), (#10083-24-6 abcam, Cambridge, UK) was diluted in DMSO following manufacturer's instructions. BMDCs were incubated with PC at a concentration of 0.5 μM for 1 h at 37°C and 5% CO_2_.

### Dendritic Cell-T Cell Co-Culture Assay

Bone marrow cells were differentiated as described above. BMDCs were seeded at a concentration of 2 × 10^5^ cells/ml in a 96-well plate and were stimulated with EndoGrade® ovalbumin (OVA, 0.3 mg/ml, Hyglos, Bernried, Germany) in the presence or absence of *L. brevis* S-layer (5 and 10 μg/ml) at 37°C and 5% CO_2_ for 24 h. T cells were isolated from spleens of 8–14 week old OT-II transgenic mice via magnetic-activated cell sorting (Pan T Cell Isolation Kit II mouse, Miltenyi Biotec). Purified T cells were adjusted to a BMDC/T cell ratio of 1:5 and co-cultured with BMDCs at 37°C and 5% CO_2_ overnight.

### qRT-PCR

To analyze Mincle mRNA expression, BMDCs from WT, Syk^fl/fl^, Syk^wt/wt^ and CARD9^−/−^ mice were seeded in a 6-well plate at 1 × 10^6^ cells/well and treated with S-layer at 10 μg/ml. Cells were collected after 3, 6, and 24 h of incubation at 37°C and 5% of CO_2_. To analyze cytokine mRNA expression, BMDCs from WT and Mincle-deficient mice were used and the same procedure was followed, except for treatment with S-layer for 12 h at final concentrations of 5 and 10 μg/ml. After stimulation, cells were centrifugated at 300xg for 5 min and washed with PBS. Next, 750 μl QIAzol was added and total RNA was isolated with the RNeasy extraction kit (Qiagen, Hilden, Germany) according to manufacturer's instructions. For qRT-PCR the One-Step-RT-PCR kit (Qiagen, Hilden, Germany) was used with an amount of RNA template of 25 ng per sample. Expression levels were measured using the AriaMx Real Time PCR system (Agilent Technologies, Santa Cruz, CA, USA). For Mincle expression, the housekeeping gene 18S rRNA was used for normalization, whereas for cytokine expression, the GADPH housekeeping gene was used.

### PCR

To analyze Syk mRNA expression, unstimulated BMDCs from WT mice, tamoxifen-treated Syk^wt/wt^ and Syk^fl/fl^ were collected and total RNA was isolated as described above. To perform the PCR, the OneStep-RT-PCR kit (Qiagen) was used with 60 ng of RNA template per sample and the following primer pair: forward 5′-TTTGGCAACATCACCCGGGAA-3′ and reverse 5′-CAGGCTTTGGGAAGGAGTAGG-3′ (Genbank accession number U25685.1). To visualize the PCR products, they were separated by agarose gel electrophoresis and visualized using GelRed® (Biotium, Fremont, CA, USA).

### Cytokine ELISAs

Culture supernatants collected after S-layer stimulation of BMDCs or the BMDC/T cell co-culture were analyzed for the pro-inflammatory cytokines IL-6 and TNF (DuoSet ELISAkits, R&D Systems, Minneapolis, MN, USA), the anti-inflammatory cytokines IL-10 (ABTS ELISA Development Kit, PeproTech, Hamburg, Germany) and TFG-β (DuoSet ELISAkits, R&D Systems, Minneapolis, MN, USA) and the T cell secreted cytokines IL-17 and IFN-γ (ABTS ELISA Development Kit, PeproTech, Hamburg, Germany) according to the manufacturer's instructions. Plates were developed with the substrate 3,3′,5,5′-Tetramethylbenzidine (TMB) and the color reaction was stopped with 2 M sulfuric acid. Absorbance was measured at 450 nm with a wavelength correction at 570 nm using a MultiskanGo microplate spectrophotometer.

### ELISpot

IFN-γ ELISpot was performed as indicated by the manufacturer (Mabtech, Stockholm, Sweden). Briefly, activated PVDF ELISpot plates (Mabtech, Stockholm, Sweden) were coated with the anti-IFN-γ coating antibody (10 μg/ml in PBS) and incubated overnight at 4°C. Excess antibody was removed followed by extensive washes with PBS. After blocking with culture medium containing 10% FBS, BMDCs stimulated with S-layer for 24 h were added to purified OT-II T cells at a ratio of 1:5 and the plate was placed in a 37°C humidified incubator with 5% CO_2_ for 24 h. After extensive washing, biotinylated anti-IFN-γ detection antibody was added and incubated for 2 h at RT. Streptavidin-HRP in PBS/0.5% FCS was added and incubated for 1 h at RT. Spots were developed with substrate solution [1,25 mM 3,3′,5,5′-Tetramethylbenzidine (TMB), 0.1 M citrate buffer, pH 6, 0.04% H_2_O_2_]. Spots were counted with an ImmunoSpot® S6 Ultimate reader (CTL) (Immunospot, Cleveland, USA) and the results were analyzed by the ImmunoSpot® SOFTWARE.

### Statistical Analysis

**Figure 1:**
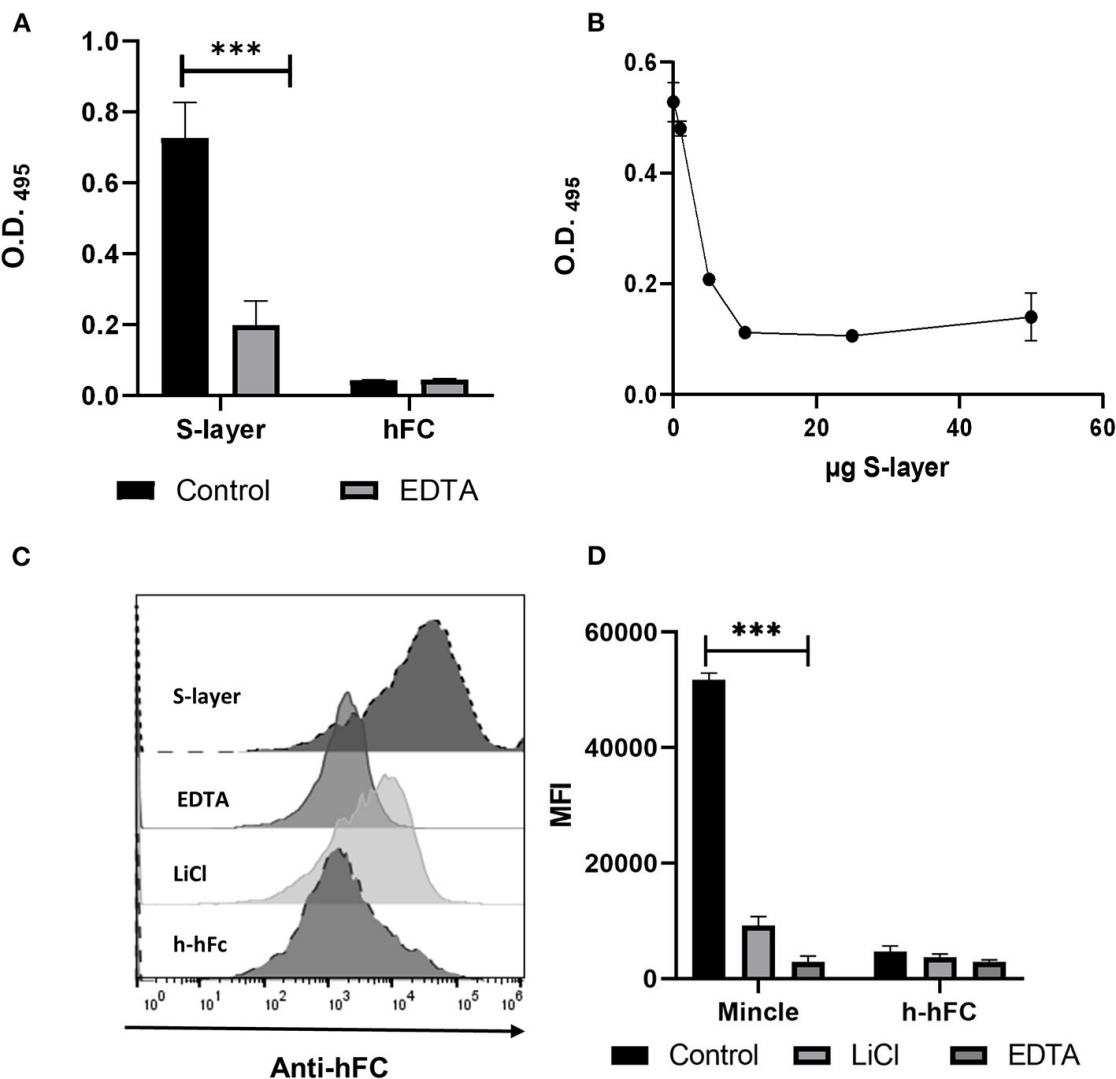
Binding of S-layer to Mincle. **(A)** S-layer from *L. brevis* (5 μg per well, diluted in PBS) was immobilized on ELISA plates and incubated with murine Mincle-hFc fusion protein (4 μg/mL) diluted either in lectin binding buffer or EDTA-containing buffer (10 mM EDTA) to analyze the Ca^2+^ dependency of the interaction. Purified hFc was used as control. Binding was measured at O.D 495. **(B)** Mincle-hFc (4 μg/mL, diluted in lectin binding buffer) was pre-incubated with increasing amounts of *L. brevis* S-layer (0–60 μg) and subsequently incubated with plate-bound TDM (50 μg/mL). Data shown are representative of three independent experiments (triplicates each). Binding was measured at O.D 495 **(C)** Binding of Mincle-hFc to *L. brevis was* analyzed by flow cytometry. *L. brevis* was treated with LiCl to remove the S-layer from the cell wall. Mincle-hFc fusion protein (4 μg/mL) was incubated with *L. brevis* (1 × 10^7^ cells/mL) either in lectin binding buffer or EDTA-containing buffer. A representative histogram plot of one binding experiment is shown. **(D)** Statistical analysis of the flow cytometry-based binding assay showing the mean fluorescence intensity (MFI) of a representative experiment. Student's *t*-test was performed to compare binding of the Mincle-hFc fusion to the hFc control alone. Data are representative of four independent experiments (triplicates each) (***p < 0.001). Data depicted are the mean + SEM.

**Figure 2:**
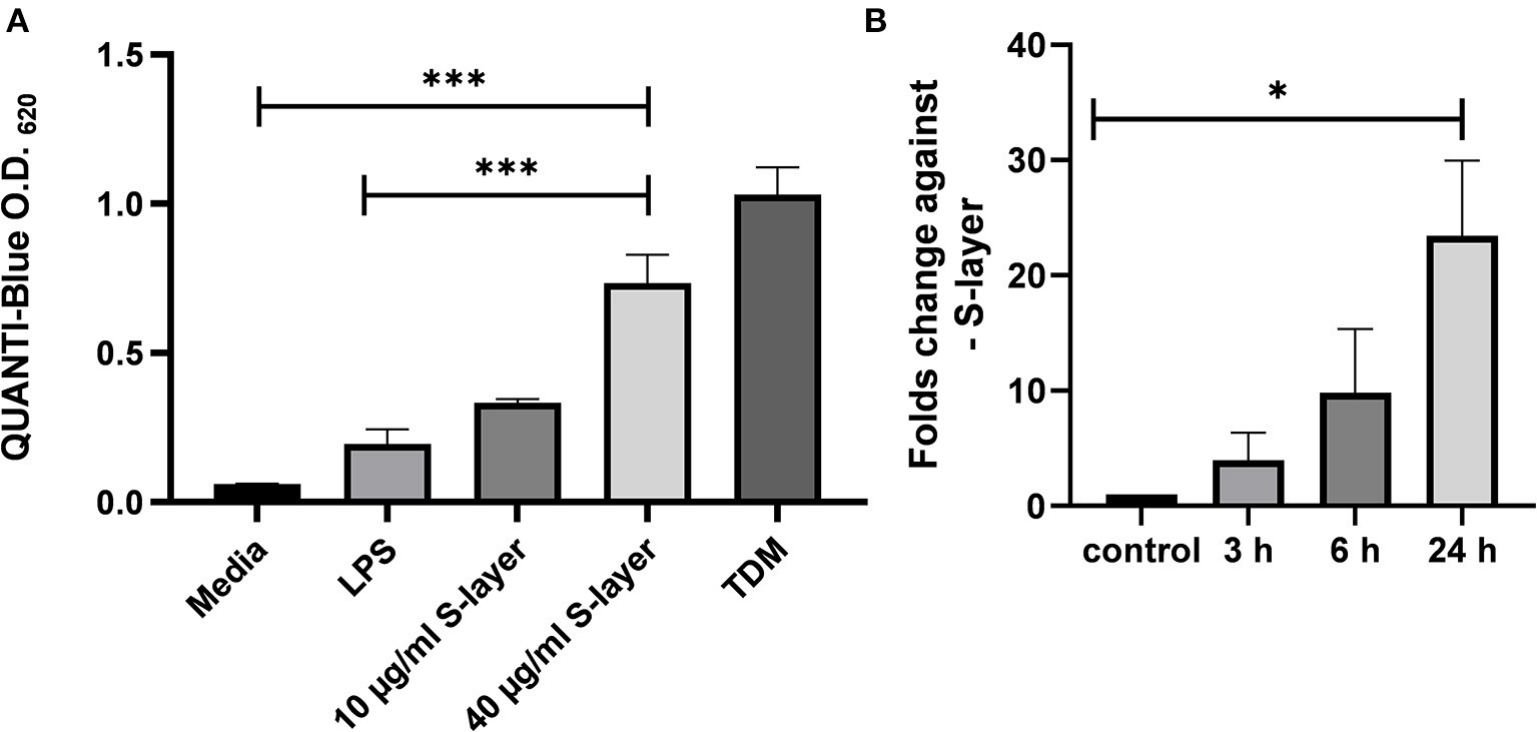
Impact of *L. brevis* S-layer on Mincle engagement and expression. **(A)** HEK cells (5 × 10^4^ cells) expressing murine Mincle (HEK-Blue™ -mMincle) coupled to a NF-κB-inducible reporter system were stimulated with *L. brevis* S-layer for 24 h. SEAP activation was determined by photometric measurement at 620 nm. **(B)** Expression levels of Mincle mRNA at different time points after incubation of BMDCs with *L. brevis* S-layer, compared to untreated cells as control. Mean + SEM are from three independent experiments. Student's *t*-test was performed to compare treatments (*p < 0.05, ***p < 0.001).

Statistical analysis was performed using the GraphPad Prism 7 software (GraphPad, San Diego, CA, USA). Data are presented as mean ± SEM for all experiments. Paired *t*-test was performed for Figures 1, 2 and two-way ANOVA with a Tukey's honest significance test was used for the remaining figures. *p* < 0.05 was considered statistically significant.

## Results

### S-Layer From *L. brevis* Is Recognized by Mincle

To analyze whether Mincle is involved in *L. brevis* S-layer recognition, we performed an ELISA-based binding assay using purified S-layer from *L. brevis* (Figure 1). The ELISA showed a significant binding of murine Mincle-hFc to *L. brevis* S-layer (Figure 1A) compared to the hFc control. Since it was previously shown that Mincle recognizes ligands in a Ca^2+^-dependent fashion (41), we determined whether the binding of Mincle-hFc to the *L. brevis* S-layer was also mediated in a Ca^2+^-dependent manner. Indeed, pre-incubation of Mincle-hFc with the chelating agent EDTA resulted in a significantly reduced binding of Mincle-hFc to S-layer (Figure 1A). To further analyze the specificity of this interaction, we performed an ELISA-based competition assay with increasing concentrations of S-layer to compete with the known Mincle ligand trehalose-6,6-dimycolate (TDM) (Figure 1B). As expected, Mincle-hFc incubation in the presence of S-layer led to reduced binding to TDM, thus confirming the specificity of the S-layer/Mincle interaction. Additionally, human Mincle-hFc was shown to bind in a Ca^2+^-dependent manner to the S-layer of *L.brevis* by the ELISA binding assay (Supplementary Figure 3A), which strengthens the results described above. We chose to focus our characterization of the S-layer/Mincle interaction on the murine model.

To prove that indeed the S-layer present in the cell wall of *L. brevis* was the ligand for Mincle, we performed a flow cytometric binding assay to L. *brevis* cells (Gating strategy shown in Supplementary Figures 3B,C). We pre-treated the bacteria with EDTA for Ca^2+^ complexation or alternatively with LiCl, which strips the S-layer off the cell wall of *L. brevis* (Figures 1C,D). In line with the ELISA-based binding assay, flow cytometric analysis indicated substantial binding of Mincle-hFc to *L. brevis* in a Ca^2+^-dependent manner. In addition, pre-treatment of *L. brevis* with LiCl led to a markedly reduced Mincle-hFc binding. These results confirm and extend a previous study that demonstrated binding of Mincle to S-layer protein from another *Lactobacillus* species, *L. kefiri* (32).

### Engagement of Mincle by S-Layer From *L. brevis*

To analyze whether *L. brevis* S-layer acts as an agonistic Mincle ligand, HEK cells expressing murine Mincle (HEK-Blue™-mMincle) were treated with *L. brevis* S-layer in different concentrations. Upon engagement, Mincle activates the Fc receptor γ-chain (FcRγ), which triggers signaling that finally leads to NF-κB activation and the induction of the secreted embryonic alkaline phosphatase (SEAP) reporter gene. SEAP activity was then detected based on the conversion of the HEK-Blue chromogenic substrate. Indeed, *L. brevis* S-layer activated the Mincle reporter cells in a concentration-dependent manner (Figure 2A). As a positive control, TDM was added to the reporter cells, also triggering SEAP induction as expected. As a specificity control, the TLR-4 agonist LPS was added to the reporter cells that did not induce significant activation. To analyze whether *L. brevis* S-layer stimulation induces Mincle expression in BMDCs, we determined Mincle mRNA expression by qRT-PCR. We observed a S-layer dependent upregulation of Mincle mRNA expression after 3, 6, and 24 h of incubation (Figure 2B). These results suggest a potential role of the S-layer/Mincle interaction for DC effector functions.

### S-Layer From *L. brevis* Differentially Induces Pro- and Anti-inflammatory Cytokines in BMDCs in a Mincle-Dependent Manner

**Figure 3:**
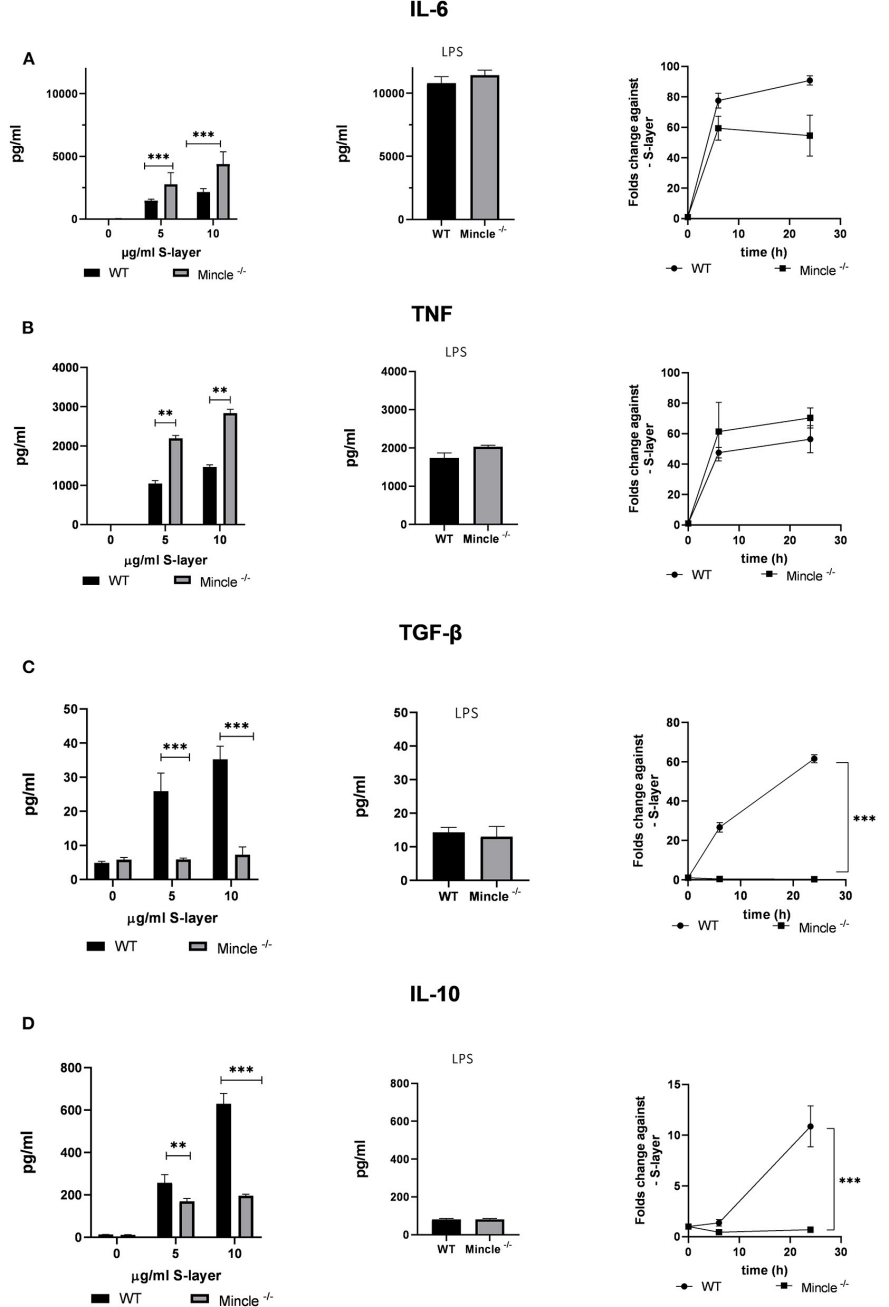
Impact of *L. brevis* S-layer on cytokine expression by BMDCs. BMDCs from WT or Mincle^−/−^ mice were stimulated with *L. brevis* S-layer for the indicated times. **(A,B)** ELISA measurement and qRT-PCR for the determination of the pro-inflammatory cytokines IL-6 and TNF on the protein and mRNA level, respectively. LPS was added as a specificity control. **(C,D)** ELISA measurement and qRT-PCR for the determination of the anti-inflammatory cytokines IL-10 and TGF-β on the protein and mRNA level, respectively. A two-way ANOVA with a Tukey's honest significance test was performed. Mean + SEM are from three independent experiments (**p < 0.01, ***p < 0.001).

Next, we examined the functional role of S-layer recognition by Mincle in BMDCs. For this purpose, we determined the effect of S-layer stimulation on cytokine expression on the mRNA and protein level in BMDCs from WT and Mincle-deficient mice. To this end, WT and Mincle^−/−^ BMDCs were stimulated with different concentrations of S-layer, whereas the TLR-4 agonist LPS was used as specificity control. Levels of both, pro- (TNF, IL-6) and anti-inflammatory cytokines (IL-10, TGF-β) were increased upon *L. brevis* S-layer stimulation in WT cells, while levels of TNF and IL-6 were markedly higher compared to IL-10 and TGF-β in response to S-layer treatment (Figures 3A–D). Surprisingly, in Mincle^−/−^ BMDCs, the outcome upon *L. brevis* S-layer stimulation differed between pro- and anti-inflammatory cytokines. While the secretion of the pro-inflammatory cytokines IL-6 and TNF was significantly increased in Mincle^−/−^ BMDCs (Figures 3A,B), both mRNA expression and secretion of the anti-inflammatory cytokines IL-10 and TGF-β were markedly decreased compared to WT BMDCs (Figures 3C,D). We also determined the expression of co-stimulatory molecules (CD40, CD80, CD86) by flow cytometry, but observed no significant differences between WT and Mincle^−/−^ BMDCs upon *L. brevis* S-layer stimulation (data not shown). These results indicate the crucial role of Mincle in provoking an anti-inflammatory response upon *L. brevis* S-layer recognition, thus highlighting the regulatory role of Mincle in DC effector functions.

### Roles of the Adaptor Protein CARD9 and the Syk Kinase in the S-Layer/Mincle Interaction

**Figure 4:**
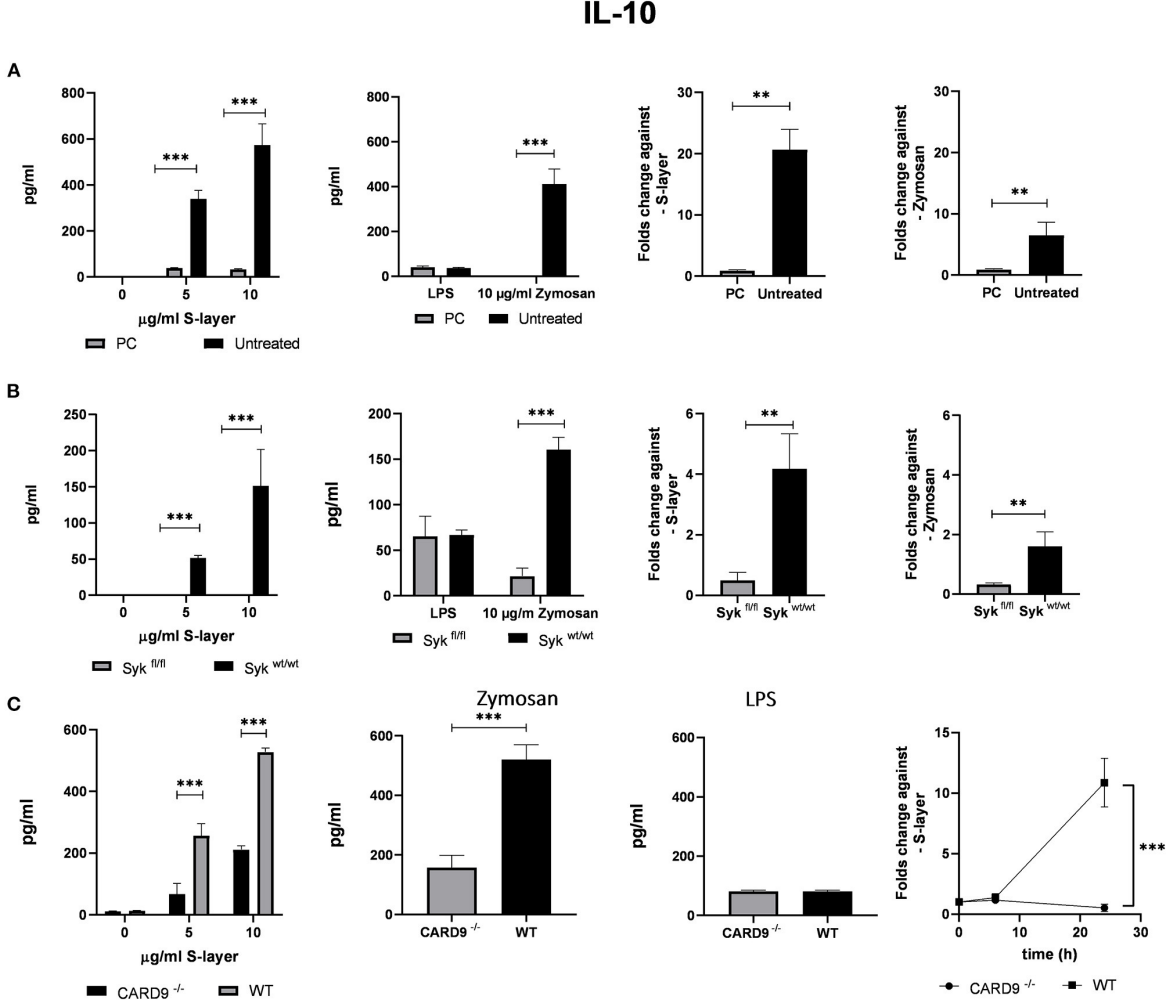
Roles of the adaptor proteins CARD9 and Syk. **(A)** BMDCs from WT mice were stimulated with S-layer from *L. brevis* in the presence or absence of the Syk inhibitor piceatannol (PC). IL-10 secretion (by ELISA) and IL-10 mRNA expression (by qRT-PCR) was measured. **(B)** BMDCs from Syk^fl/fl^ and Syk^wt/wt^ mice were treated with 4-hydroxytamoxifen to conditionally knock-out Syk expression and were incubated with *L. brevis* S-layer. IL-10 secretion (by ELISA) and IL-10 mRNA expression (by qRT-PCR) were measured. **(C)** BMDCs from CARD9^−/−^ mice were incubated with *L. brevis* S-layer and the secretion and mRNA expression of IL-10 were measured. A two-way ANOVA with a Tukey's honest significance test was performed. Mean + SEM are from three independent experiments (**p < 0.01, ***p < 0.001).

To analyze the contribution of Mincle-mediated signaling to cytokine production induced by *L. brevis* S-layer, we targeted two proteins that play a crucial role in the signaling pathway of Mincle, the spleen tyrosine kinase Syk and the adaptor protein CARD9 (42–44). To evaluate Syk involvement in the Mincle-mediated response to *L. brevis* S-layer stimulation, we employed two approaches, pharmacological inhibition and genetic ablation of Syk and/or CARD9. First, we used the Syk inhibitor piceatannol (PC) which is a Syk-selective tyrosine kinase inhibitor (45). This compound was previously reported to inhibit the Syk signaling pathway downstream of Mincle (46, 47). Due to the crucial role of Mincle in the production of anti-inflammatory cytokines after *L. brevis* S-layer stimulation (Figures 3C,D), we focused on the immune modulatory cytokine IL-10. Indeed, IL-10 mRNA expression and IL-10 secretion by BMDCs were almost completely abolished upon pre-treatment with PC, thus showing the importance of Syk in the signaling pathway leading to IL-10 expression on the mRNA and protein level (Figure 4A). As positive control, we used the Dectin-1 ligand depleted zymosan. Depleted Zymosan was obtained by treating zymosan with hot alkali to abrogate its Toll-like receptor (TLR)-stimulating properties. Hence, depleted zymosan activates the CLR Dectin-1, but not TLR2. This ligand was previously reported to trigger IL-10 secretion by Dectin-1 ligation in a Syk-dependent fashion (48).

Our second approach was the use of a conditional Syk-deficient mouse line. BMDCs from the Syk^fl/fl^ and the respective control Syk^wt/wt^ were cultured and 4-hydroxytamoxifen was added to the cells for a conditional Syk knock-out. The results of this approach corroborated the findings obtained for PC treatment since a markedly reduced IL-10 mRNA and protein expression was found in Syk^fl/fl^ BMDCs compared to the control Syk^wt/wt^ BMDCs (Figure 4B). Our findings indicate that Syk is critically involved in Mincle-mediated IL-10 production after *L. brevis* S-layer incubation.

To elucidate the contribution of the adapter protein CARD9 to Mincle-mediated signaling, we used BMDCs obtained from CARD9^−/−^ mice (49), and performed qRT-PCR and cytokine ELISA measurements to evaluate IL-10 mRNA expression and cytokine secretion after incubation with *L. brevis* S-layer. Simultaneously, we corroborated by qRT-PCR that the expression of Mincle was not altered in the CARD9^−/−^ and Syk^fl/fl^ BMDCs (Supplementary Figure 4) Consistently, IL-10 mRNA expression and secretion was largely CARD9-dependent since CARD9 deficiency markedly inhibited IL-10 production (Figure 4C).

### *L. brevis* S-Layer/Mincle Interaction Affects T Cell Cytokine Expression

**Figure 5:**
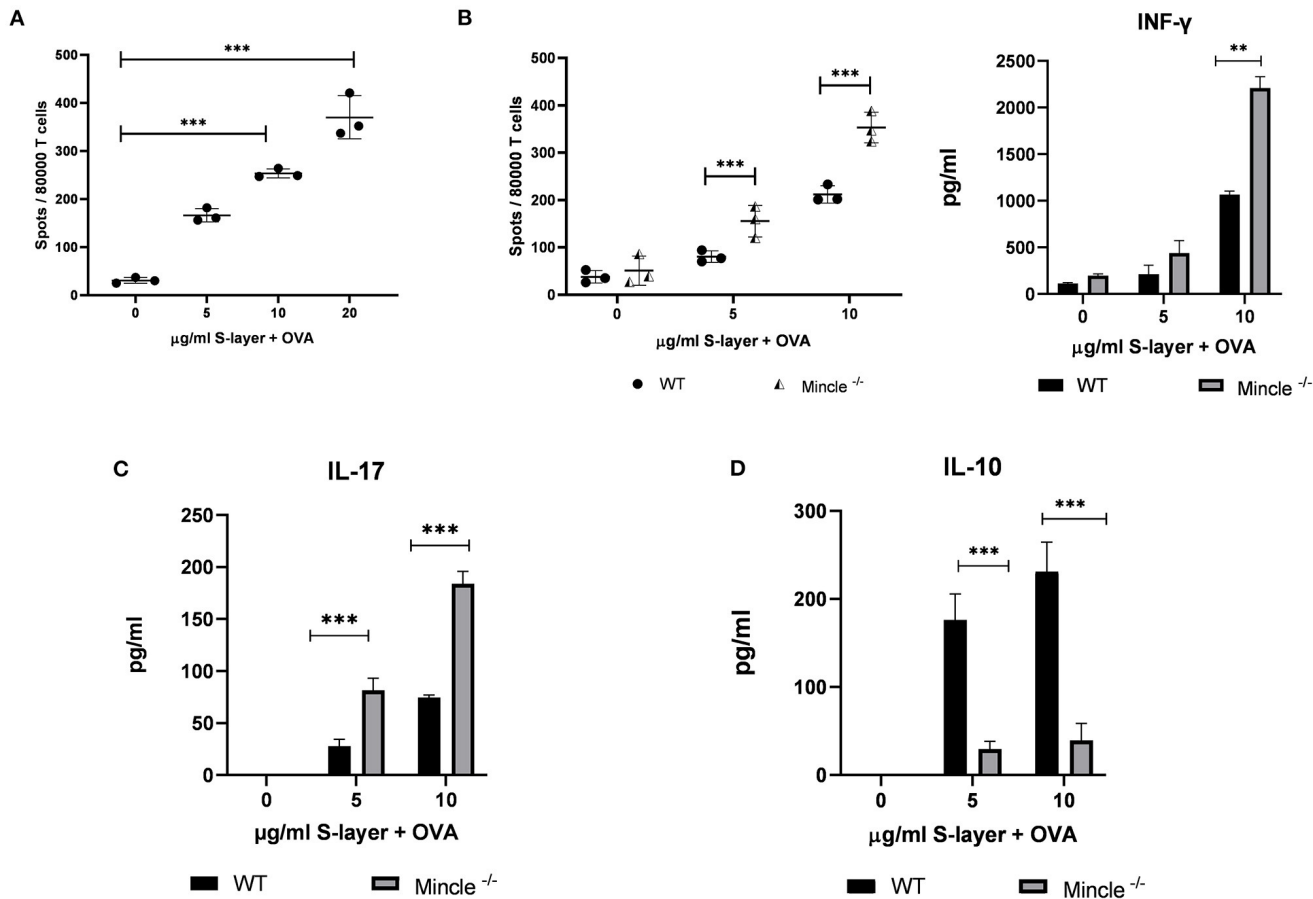
Impact of the *L. brevis* S-layer/Mincle interaction on T cell cytokine. BMDCs from WT or Mincle^−/−^ mice were co-cultured with T cells from OT-II transgenic mice and were stimulated with 0.3 mg/ml ovalbumin (OVA) in the presence or absence of *L. brevis* S-layer for 24 h. **(A)** ELISpot measurement of the number of IFN-γ-producing OT-II T cells co-cultured with BMDCs from WT mice upon stimulation with OVA/*L. brevis* S-layer. **(B)** ELISpot and ELISA measurement of the number of IFN-γ-producing T cells and IFN-γ levels in the culture supernatant, respectively, after co-culture of WT or Mincle^−/−^ BMDCs with OT-II T cells in the presence of OVA and different concentrations of *L. brevis* S-layer. **(C,D)** ELISA measurement of IL-17 production **(C)** and IL-10 production **(D)**, after co-culture of WT or Mincle^−/−^ BMDCs with OT-II T cells in the presence of OVA and different concentrations of *L. brevis* S-layer. A two-way ANOVA with a Tukey's honest significance test was performed. Mean ± SEM are shown from two independent experiments (**p < 0.01, ***p < 0.001).

To analyze whether the S-layer/Mincle interaction in BMDCs affected their T cell activating capacity and subsequent T cell effector functions, we co-cultured BMDCs from Mincle^−/−^ or WT control mice with T cells from OT-II mice. OT-II T cells have a transgenic T cell receptor specific for the OVA_323−339_ peptide presented by the MHC-II molecule I-A^b^ (50). To assess the impact of the S-layer/Mincle interaction on CD4^+^ T cell priming, IFN-γ production by OT-II T-cells was measured by ELISA and ELISpot. Upon OVA stimulation alone, only marginal IFN-γ production and a low frequency of IFN-γ^+^ T cells was detected (Figures 5A,B). However, when BMDCs were incubated with OVA in the presence of *L. brevis* S-layer, the number of IFN-γ-producing T cells increased in a concentration-dependent manner (Figure 5A). Interestingly, we detected a significant increase of the number of IFN-γ-producing T cells as well as secreted IFN-γ levels, when Mincle^−/−^ BMDCs were used to prime OT-II T cells in the presence of *L. brevis* S-layer, compared to WT BMDCs (Figure 5B). Similar results were obtained for IL-17 production (Figure 5C). In contrast, IL-10 production was markedly decreased when Mincle^−/−^ BMDCs were used to stimulate OT-II T cells in the presence of S-layer (Figure 5D). These findings clearly demonstrate the crucial role of Mincle in the balance of pro- and anti-inflammatory responses upon *L. brevis* S-layer recognition and highlights its role in T cell priming and the modulation of initiated CD4^+^ T cell responses.

## Discussion

In the past years, more and more researchers have pointed out the very important role of the gut microbiome in human health (51, 52). An imbalance of microbes in the intestines may contribute not only to local bowel-related diseases, but to a wide range of disorders affecting even distant organs such as brain and lung (12). Several approaches demonstrated the role of microbiota in immune homeostasis and the development of the immune system (53).

**Figure 6:**
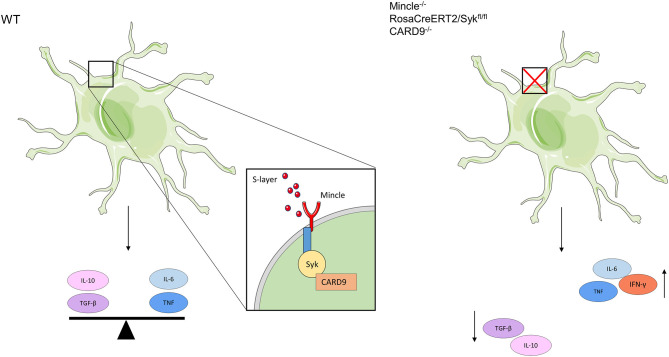
Schematic depiction showing the functional role of the *L. brevis* S-layer/Mincle interaction in BMDCs. When *L. brevis* S-layer is recognized by Mincle, this interaction triggers the production of both pro- (IL-6 and TNF) and anti-inflammatory (IL-10 and TGF-β) cytokines, thus creating a balance in the cytokine response in a Syk/CARD9-dependent fashion. When the S-layer recognition by Mincle is abrogated or Mincle-mediated pathways are inhibited, BMDCs fail to maintain this balance in cytokine production. As a consequence, the levels of pro-inflammatory cytokines are markedly increased, whereas the production of anti-inflammatory cytokines is decreased. This also affects subsequent T cell priming and effector functions; thus, it may impact immune homeostasis.

In this study, we identified the S-layer from *L. brevis* as a ligand of the CLR Mincle. This observation confirms and extends findings of a previous study that identified Mincle as a receptor of S-layer protein from another *Lactobacillus* species, *L. kefiri* (32). We demonstrate that Mincle interacts with purified S-layer from *L. brevis* in a Ca^2+^-dependent manner. This interaction triggers a Syk-CARD9-dependent induction of both pro- and anti-inflammatory cytokines. Interestingly, we observed a major impact of Mincle signaling on the production of immune-modulatory cytokines, particularly IL-10. Previous data suggested that probiotic lactobacilli impair the recognition of pathogen-associated molecular patterns (PAMPs) (54), and alter the production of pro/anti-inflammatory cytokines, thus modulating inflammation. Whereas, previous studies focused mainly on the immune stimulatory role of Mincle (23, 30, 32), our study highlights its regulatory role in modulating effector functions of DCs (see a summary in Figure 6).

Recent studies point out the importance of Mincle in the regulation of human gut homeostasis (3, 12). The induction of both, pro- and anti-inflammatory cytokines, is already known for S-layers of other bacterial species (25, 54). In this study, we have elucidated the contribution of the Mincle/Syk/CARD9 axis to the *L. brevis*-mediated modulation of DC cytokine production.

The interaction of Mincle with microbiota and especially with *Lactobacillus sp*. is important for IL-17 and IL-22 production (3). Deficiency in the production of these cytokines correlated with increased peripheral dissemination of commensal bacteria. This bacterial translocation was accompanied by an increased liver infiltration of neutrophils and monocytes in Mincle-deficient mice (55). A recent elegant study demonstrated that DCs sense mucosa-associated bacteria via the Mincle/Syk axis, thus triggering the production of IL-6 and IL-23, i.e., cytokines that regulate IL-22 and IL-17 production by T cells (3). This interaction promotes the intestinal immune barrier, limits microbial translocation and prevents systemic inflammation and its metabolic consequences.

CARD9 is involved in gut homeostasis and plays a crucial role in the development of diseases such as inflammatory bowel disease (IBD) and colorectal cancer (56). Polymorphisms in CARD9 are key risk factors for IBD development, indicating that CARD9 signaling is critical for intestinal immune homeostasis (57). A mutation in CARD9 provokes a disbalance in the composition and functions of the gut microbiota, finally leading to intestinal inflammation (56). In this study, we found that interference with the S-layer/Mincle interaction by targeting the Syk/CARD9 pathway created a disbalance in cytokine production by DCs. Down-regulation of the expression of the anti-inflammatory cytokine IL-10 was accompanied by an up-regulation of the pro-inflammatory cytokines IL-6 and TNF (Figure 6). This change also affected CD4^+^ T cell priming and subsequent IFN-γ production by CD4^+^ T cells.

The results of this study contribute substantially to understanding the mechanism of action of probiotics that seek to reverse and cure chronic disease not only in the gut environment, but systemically. Future probiotics may target specific human disease states, including effective prophylactics that reduce the incidence of infectious disease in at risk populations. This study helps to shed light on how microbiota may be involved in balancing immune homeostasis.

## Data Availability Statement

The raw data supporting the conclusions of this article will be made available by the authors, without undue reservation.

## Ethics Statement

Sacrificing of mice for scientific purposes was approved by the Animal Officers of the University of Veterinary Medicine Hannover (TiHo-T-2019-7, TiHo-T-2019-13).

## Author Contributions

MPA and BL contributed to the conception and design of the study. MPA and GG-D carried out the experimental procedure. JS, AD, and JR contributed essential reagents and revised the manuscript. MPA wrote the initial version of the manuscript. GG-D and BL revised the manuscript. All authors contributed to the article and approved the submitted version.

## Conflict of Interest

The authors declare that the research was conducted in the absence of any commercial or financial relationships that could be construed as a potential conflict of interest.

## References

[1] Pickard JM Zeng MY Caruso R Núñez G. Gut microbiota: role in pathogen colonization, immune responses and inflammatory disease. Immunol Rev. (2017) 279:70–89. 10.1111/imr.12567 28856738 PMC5657496

[2] Rinninella E Raoul P Cintoni M Franceschi F Miggiano GAD Gasbarrini A. What is the healthy gut microbiota composition? A changing ecosystem across age, environment, diet, and diseases. Microorganisms. (2019) 7:14. 10.3390/microorganisms7010014 30634578 PMC6351938

[3] Martinez-Lopez M Iborra S Conde-Garrosa R Mastrangelo A Danne C Mann ER. Microbiota sensing by Mincle-Syk axis in dendritic cells regulates interleukin-17 and−22 production and promotes intestinal barrier integrity. Immunity. (2019) 50:446–61.e9. 10.1016/j.immuni.2018.12.020 30709742 PMC6382412

[4] Souza DG Vieira AT Soares AC Pinho V Nicoli JR Vieira LQ. The essential role of the intestinal microbiota in facilitating acute inflammatory responses. J Immunol. (2004) 173:4137–46. 10.4049/jimmunol.173.6.4137 15356164

[5] Maslowski KM Vieira AT Ng A Kranich J Sierro F Yu D. Regulation of inflammatory responses by gut microbiota and chemoattractant receptor GPR43. Nature. (2009) 461:1282–6. 10.1038/nature08530 19865172 PMC3256734

[6] Abt MC Osborne LC Monticelli LA Doering TA Alenghat T Sonnenberg GF. Commensal bacteria calibrate the activation threshold of innate antiviral immunity. Immunity. (2012) 37:158–70. 10.1016/j.immuni.2012.04.011 22705104 PMC3679670

[7] Ruane D Chorny A Lee H Faith J Pandey G Shan M. Microbiota regulate the ability of lung dendritic cells to induce IgA class-switch recombination and generate protective gastrointestinal immune responses. J Exp Med. (2016) 213:53–73. 10.1084/jem.20150567 26712806 PMC4710201

[8] Honda K Littman DR. The microbiome in infectious disease and inflammation. Annu Rev Immunol. (2012) 30:759–95. 10.1146/annurev-immunol-020711-074937 22224764 PMC4426968

[9] Iida N Dzutsev A Stewart CA Smith L Bouladoux N Weingarten RA. Commensal bacteria control cancer response to therapy by modulating the tumor microenvironment. Science. (2013) 342:967–70. 10.1126/science.1240527 24264989 PMC6709532

[10] Rakoff-Nahoum S Hao L Medzhitov R. Role of toll-like receptors in spontaneous commensal-dependent colitis. Immunity. (2006) 25:319–29. 10.1016/j.immuni.2006.06.010 16879997

[11] Wu H-J Ivanov II Darce J Hattori K Shima T Umesaki Y. Gut-residing segmented filamentous bacteria drive autoimmune arthritis via T helper 17 cells. Immunity. (2010) 32:815–27. 10.1016/j.immuni.2010.06.001 20620945 PMC2904693

[12] Negi S Pahari S Bashir H Agrewala JN. Gut microbiota regulates Mincle mediated activation of lung dendritic cells to protect against *Mycobacterium tuberculosis*. Front Immunol. (2019) 10:1142. 10.3389/fimmu.2019.01142 31231363 PMC6558411

[13] Ganal SC Sanos SL Kallfass C Oberle K Johner C Kirschning C. Priming of natural killer cells by nonmucosal mononuclear phagocytes requires instructive signals from commensal microbiota. Immunity. (2012) 37:171–86. 10.1016/j.immuni.2012.05.020 22749822

[14] Khosravi A Yanez A Price JG Chow A Merad M Goodridge HS. Gut microbiota promote hematopoiesis to control bacterial infection. Cell Host Microbe. (2014) 15:374–81. 10.1016/j.chom.2014.02.006 24629343 PMC4144825

[15] Ichinohe T Pang IK Kumamoto Y Peaper DR Ho JH Murray TS. Microbiota regulates immune defense against respiratory tract influenza A virus infection. Proc Natl Acad Sci USA. (2011) 108:5354–9. 10.1073/pnas.1019378108 21402903 PMC3069176

[16] Deering KE Devine A O'Sullivan TA Lo J Boyce MC Christophersen CT. Characterizing the composition of the pediatric gut microbiome: a systematic review. Nutrients. (2019) 12:16. 10.3390/nu12010016 31861722 PMC7019424

[17] Dunne C O'Mahony L Murphy L Thornton G Morrissey D O'Halloran S. *In vitro* selection criteria for probiotic bacteria of human origin: correlation with *in vivo* findings. Am J Clin Nutr. (2001) 73:386S−92S. 10.1093/ajcn/73.2.386s 11157346

[18] Mohamadzadeh M Duong T Hoover T Klaenhammer TR. Targeting mucosal dendritic cells with microbial antigens from probiotic lactic acid bacteria. Exp Rev Vaccines. (2008) 7:163–74. 10.1586/14760584.7.2.163 18324887

[19] Piqué N Berlanga M Miñana-Galbis D. Health benefits of heat-killed (tyndallized) probiotics: an overview. Int J Mol Sci. (2019) 20:2534. 10.3390/ijms20102534 31126033 PMC6566317

[20] Hill C Guarner F Reid G Gibson GR Merenstein DJ Pot B. The International Scientific Association for Probiotics and Prebiotics consensus statement on the scope and appropriate use of the term probiotic. Nat Rev Gastroenterol Hepatol. (2014) 11:506–14. 10.1038/nrgastro.2014.66 24912386

[21] Hynönen U Palva A. Lactobacillus surface layer proteins: structure, function and applications. Appl Microbiol Biotechnol. (2013) 97:5225–43. 10.1007/s00253-013-4962-2 23677442 PMC3666127

[22] Suzuki S Yokota K Igimi S Kajikawa A. Comparative analysis of immunological properties of S-layer proteins isolated from *Lactobacillus* strains. Microbiology. (2019) 165:188–96. 10.1099/mic.0.000766 30620267

[23] Konstantinov SR Smidt H Vos WM De Bruijns SCM Kaur S Valence F. S layer protein A of *Lactobacillus acidophilus* NCFM regulates immature dendritic cell and T cell functions. Proc Natl Acad Sci USA. (2008) 105:19474–9. 10.1073/pnas.0810305105 19047644 PMC2592362

[24] Prado Acosta M Geoghegan EM Lepenies B Ruzal S Kielian M Martinez MG. Surface (S) layer proteins of *Lactobacillus acidophilus* block virus infection via DC-SIGN interaction. Front Microbiol. (2019) 10:810. 10.3389/fmicb.2019.00810 31040840 PMC6477042

[25] Chinthamani S Settem RP Honma K Kay JG Sharma A. Macrophage inducible C-type lectin (Mincle) recognizes glycosylated surface (S)-layer of the periodontal pathogen Tannerella forsythia. PLoS ONE. (2017) 12:e0173394. 10.1371/journal.pone.0173394 28264048 PMC5338828

[26] Mayer S Raulf M-K Lepenies B. C-type lectins: their network and roles in pathogen recognition and immunity. Histochem Cell Biol. (2017) 147:223–237. 10.1007/s00418-016-1523-7 27999992

[27] Prado Acosta M Lepenies B. Bacterial glycans and their interactions with lectins in the innate immune system. Biochem Soc Trans. (2019) 47:1569–79. 10.1042/BST20170410 31724699

[28] Li T-H Liu L Hou Y-Y Shen S-N Wang T-T. C-type lectin receptor-mediated immune recognition and response of the microbiota in the gut. Gastroenterol Rep. (2019) 7:312–21. 10.1093/gastro/goz028 31687150 PMC6821170

[29] Prado Acosta M Ruzal SM Cordo SM. S-layer proteins from *Lactobacillus* sp. inhibit bacterial infection by blockage of DC-SIGN cell receptor. Int J Biol Macromol. (2016) 92:998–1005. 10.1016/j.ijbiomac.2016.07.096 27498415

[30] Gao X Huang L Zhu L Mou C Hou Q Yu Q. Inhibition of H9N2 virus invasion into dendritic cells by the S-Layer protein from *L*. acidophilus ATCC 4356. (2016) 6:1–10. 10.3389/fcimb.2016.00137 27826541 PMC5078685

[31] Lightfoot YL Selle K Yang T Goh YJ Sahay B Zadeh M. SIGNR 3-dependent immune regulation by *Lactobacillus acidophilus* surface layer protein A in colitis. EMBO J. (2015) 34:881–95. 10.15252/embj.201490296 25666591 PMC4388597

[32] Malamud M Carasi P Assandri MH Freire T Lepenies B Serradell M de LA. S-layer glycoprotein from *lactobacillus kefiri* exerts its immunostimulatory activity through glycan recognition by Mincle. Front Immunol. (2019) 10:1422. 10.3389/fimmu.2019.01422 31297112 PMC6607945

[33] Abdelazez A Abdelmotaal H Evivie SE Melak S Jia F-F Khoso MH. Screening potential probiotic characteristics of *Lactobacillus brevis* strains *in vitro* and intervention effect on type I diabetes *in vivo*. Biomed Res Int. (2018) 2018:7356173. 10.1155/2018/7356173 30327780 PMC6169223

[34] Fang F Xu J Li Q Xia X Du G. Characterization of a *Lactobacillus brevis* strain with potential oral probiotic properties. BMC Microbiol. (2018) 18:221. 10.1186/s12866-018-1369-3 30577728 PMC6303927

[35] Fina Martin J Palomino MM Cutine AM Modenutti CP Fernández Do Porto DA Allievi MC. Exploring lectin-like activity of the S-layer protein of *Lactobacillus acidophilus* ATCC 4356. Appl Microbiol Biotechnol. (2019) 103:4839–57. 10.1007/s00253-019-09795-y 31053916

[36] Kostarnoy AV Gancheva PG Lepenies B Tukhvatulin AI Dzharullaeva AS Polyakov NB. Receptor Mincle promotes skin allergies and is capable of recognizing cholesterol sulfate. Proc Natl Acad Sci USA. (2017) 114:E2758–65. 10.1073/pnas.1611665114 28292894 PMC5380039

[37] Heß R Storcksdieck Genannt Bonsmann M Lapuente D Maaske A Kirschning C Ruland J. Glycosylation of HIV Env impacts IgG subtype responses to vaccination. Viruses. (2019) 11:153. 10.3390/v11020153 30781796 PMC6410111

[38] Monteiro JT Schön K Ebbecke T Goethe R Ruland J Baumgärtner W. The CARD9-associated C-type lectin, Mincle, recognizes la crosse virus (LACV) but plays a limited role in early antiviral responses against LACV. Viruses. (2019) 11:1–18. 10.3390/v11030303 30917612 PMC6466035

[39] Stockinger B Zal T Zal A Gray D. B cells solicit their own help from T cells. J Exp Med. (1996) 183:891–9. 10.1084/jem.183.3.891 8642293 PMC2192359

[40] Mayer S Moeller R Monteiro JT Ellrott K Josenhans C Lepenies B. C-Type lectin receptor (CLR)-Fc fusion proteins as tools to screen for novel CLR/bacteria interactions: an exemplary study on preselected *Campylobacter jejuni* isolates. Front Immunol. (2018) 9:213. 10.3389/fimmu.2018.00213 29487596 PMC5816833

[41] Schoenen H Bodendorfer B Hitchens K Manzanero S Werninghaus K Nimmerjahn F. Cutting edge: Mincle is essential for recognition and adjuvanticity of the mycobacterial cord factor and its synthetic analog trehalose-dibehenate. J Immunol. (2010) 184:2756–60. 10.4049/jimmunol.0904013 20164423 PMC3442336

[42] Lamas B Michel M-L Waldschmitt N Pham H-P Zacharioudaki V Dupraz L. Card9 mediates susceptibility to intestinal pathogens through microbiota modulation and control of bacterial virulence. Gut. (2018) 67:1836–44. 10.1136/gutjnl-2017-314195 28790160

[43] Malik A Sharma D Malireddi RKS Guy CS Chang T-C Olsen SR. SYK-CARD9 signaling axis promotes gut fungi-mediated inflammasome activation to restrict colitis and colon cancer. Immunity. (2018) 49:515–30.e5. 10.1016/j.immuni.2018.08.024 30231985 PMC6541497

[44] Leshchiner ES Rush JS Durney MA Cao Z Dančík V Chittick B. Small-molecule inhibitors directly target CARD9 and mimic its protective variant in inflammatory bowel disease. Proc Natl Acad Sci USA. (2017) 114:11392–7. 10.1073/pnas.1705748114 29073062 PMC5664502

[45] Seow C-J Chue S-C Wong WSF. Piceatannol, a Syk-selective tyrosine kinase inhibitor, attenuated antigen challenge of guinea pig airways *in vitro*. Eur J Pharmacol. (2002) 443:189–96. 10.1016/S0014-2999(02)01534-0 12044809

[46] Piotrowska H Kucinska M Murias M. Biological activity of piceatannol: leaving the shadow of resveratrol. Mutat Res. (2012) 750:60–82. 10.1016/j.mrrev.2011.11.001 22108298

[47] Suzuki Y Nakano Y Mishiro K Takagi T Tsuruma K Nakamura M. Involvement of Mincle and syk in the changes to innate immunity after ischemic stroke. Sci Rep. (2013) 3:1–7. 10.1038/srep03177 24212132 PMC3822396

[48] Alvarez Y Municio C Alonso S Sánchez Crespo M Fernández N. The induction of IL-10 by Zymosan in dendritic cells depends on CREB activation by the coactivators CREB-binding protein and TORC2 and autocrine PGE 2. J Immunol. (2009) 183:1471–9. 10.4049/jimmunol.0900312 19564345

[49] Gross O Gewies A Finger K Schäfer M Sparwasser T Peschel C. Card9 controls a non-TLR signalling pathway for innate anti-fungal immunity. Nature. (2006) 442:651–6. 10.1038/nature04926 16862125

[50] Barnden MJ Allison J Heath WR Carbone FR. Defective TCR expression in transgenic mice constructed using cDNA-based alpha- and beta-chain genes under the control of heterologous regulatory elements. Immunol Cell Biol. (1998) 76:34–40. 10.1046/j.1440-1711.1998.00709.x 9553774

[51] Wu H-J Wu E. The role of gut microbiota in immune homeostasis and autoimmunity. Gut Microbes. (2012) 3:4–14. 10.4161/gmic.19320 22356853 PMC3337124

[52] Pushpanathan P Mathew GS Selvarajan S Seshadri KG Srikanth P. Gut microbiota and its mysteries. Indian J Med Microbiol. (2019) 37:268–77. 10.4103/ijmm.IJMM_19_373 31745030

[53] Peterson CT Sharma V Elmén L Peterson SN. Immune homeostasis, dysbiosis and therapeutic modulation of the gut microbiota. Clin Exp Immunol. (2015) 179:363–77. 10.1111/cei.12474 25345825 PMC4337670

[54] Matsubara VH Ishikawa KH Ando-Suguimoto ES Bueno-Silva B Nakamae AEM Mayer MPA. Probiotic bacteria alter pattern-recognition receptor expression and cytokine profile in a human macrophage model challenged with *Candida albicans* and lipopolysaccharide. Front Microbiol. (2017) 8:1–12. 10.3389/fmicb.2017.02280 29238325 PMC5712552

[55] Nakamoto N Amiya T Aoki R Taniki N Koda Y Miyamoto K. Commensal Lactobacillus controls immune tolerance during acute liver injury in mice. Cell Rep. (2017) 21:1215–26. 10.1016/j.celrep.2017.10.022 29091761

[56] Lamas B Richard ML Leducq V Pham H-P Michel M-L Da Costa G. CARD9 impacts colitis by altering gut microbiota metabolism of tryptophan into aryl hydrocarbon receptor ligands. Nat Med. (2016) 22:598–605. 10.1038/nm.4102 27158904 PMC5087285

[57] Hartjes L Ruland J. CARD9 signaling in intestinal immune homeostasis and oncogenesis. Front Immunol. (2019) 10:419. 10.3389/fimmu.2019.00419 30906296 PMC6418414

