# Supplementary material for: S-Layer From Lactobacillus brevis Modulates Antigen-Presenting Cell Functions via the Mincle-Syk-Card9 Axis
Source: Front Immunol. 2021 Mar 1;12:602067. doi: 10.3389/fimmu.2021.602067 (PMC7957004; doi:10.3389/fimmu.2021.602067)
Supplement: Supplementary file 1 [file Data_Sheet_1.docx]

Supplementary Material

| **Primers** | **Forward** | **Reverse** |
| --- | --- | --- |
| IL-6 | 5´-GAGGATACCACTCCCAACAGACC - 3´ | 5´-AAGTGCATCATCGTTGTTCATACA -3´ |
| IL-10 | 5´-GGTTGCCAAGCCTTATCGGA -3´ | 5´- ACCTGCTCCACTGCCTTGCT -3´ |
| TNF | 5´- CATCTTCTCAAAATTCGAGTGACAA -3´ | 5´-TGGGAGTAGACAAGGTACAACCC- 3´ |
| TGF-β | 5´- TGACGTCACTGGAGTTGTACGG -3´ | 5´-GGTTCATGTCATGGATGGTGC-3 |
| GADPH | 5´-AGGTCGGTGTGAACGGATTTG-3´ | 5´-TGTAGACCATGTAGTTGAGGT CA-3´ |
| Mincle | ThermoFisher Scientific, Ref. Mm01183703_m1 | |
| 18S | ThermoFisher Scientific, Ref. Mm03928990_g1 | |

**Supplementary Table 1.** Primer sequences for quantitative (q)Real Time PCR


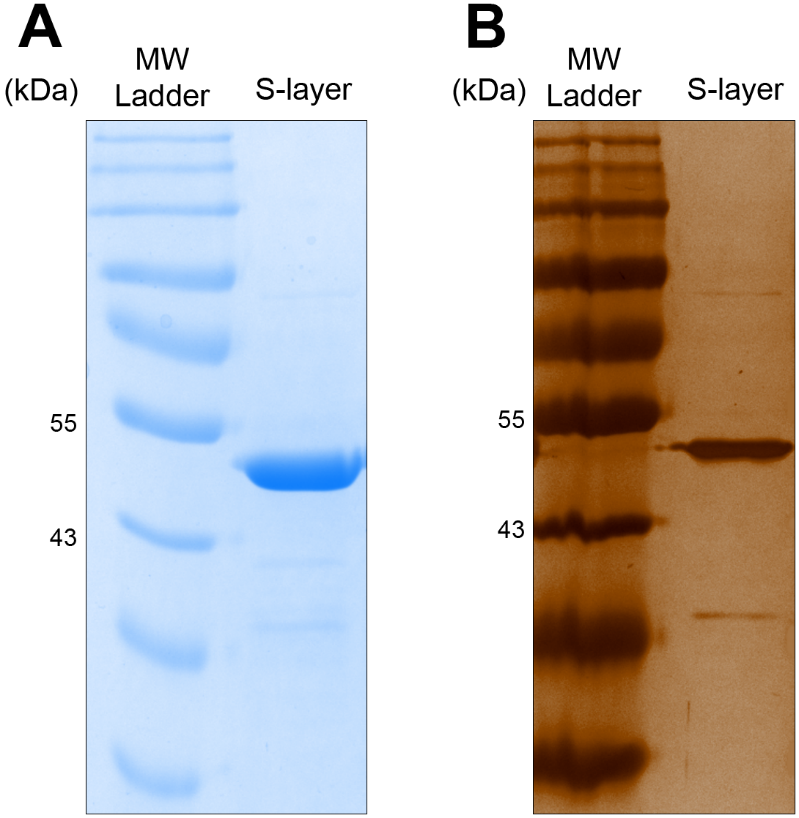


**Supplementary Figure 1.** SDS-PAGE of purified S-layer protein. (A) Coomassie blue staining. (B) Silver staining.


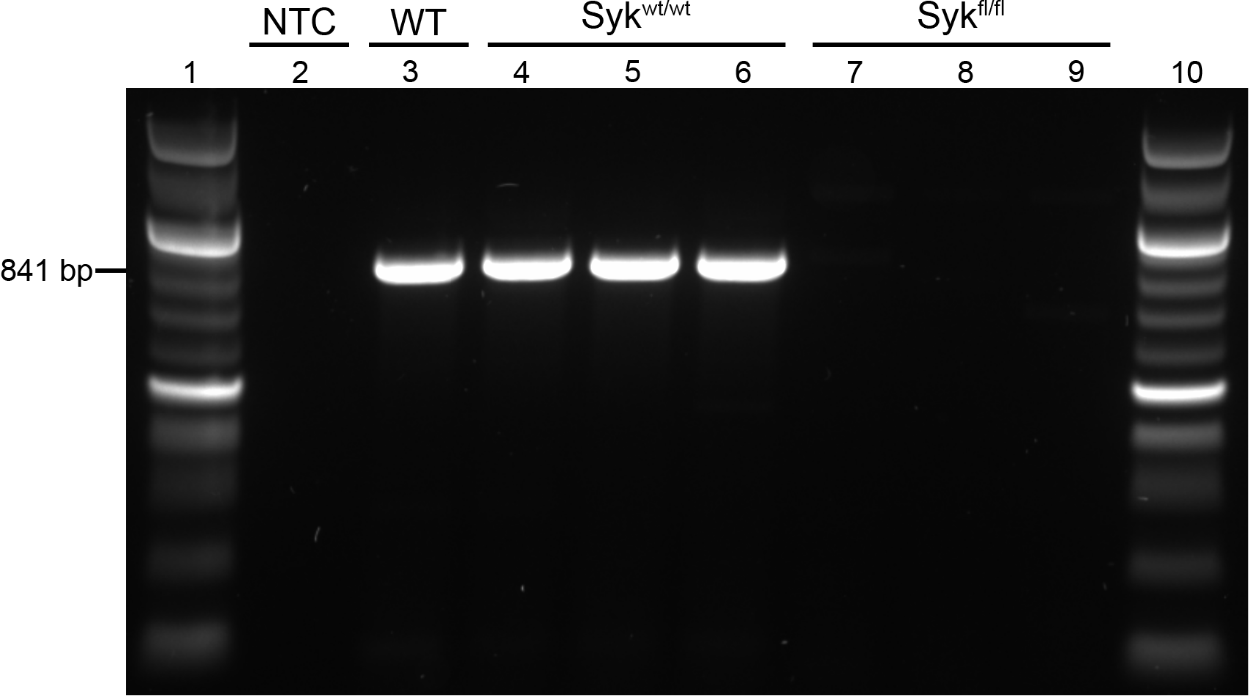


**Supplementary Figure 2.** Expression of Syk mRNA in unstimulated BMDCs. Lanes 1 & 10, 100 bp DNA ladder; lane 2, no template control (NTC); lane 3, sample from WT (C57BL/6) BMDCs; lanes 4 – 6, samples from tamoxifen-treated Syk^wt/wt^ BMDCs; lanes 7-9, samples from tamoxifen-treated Syk^fl/fl^ BMDCs.


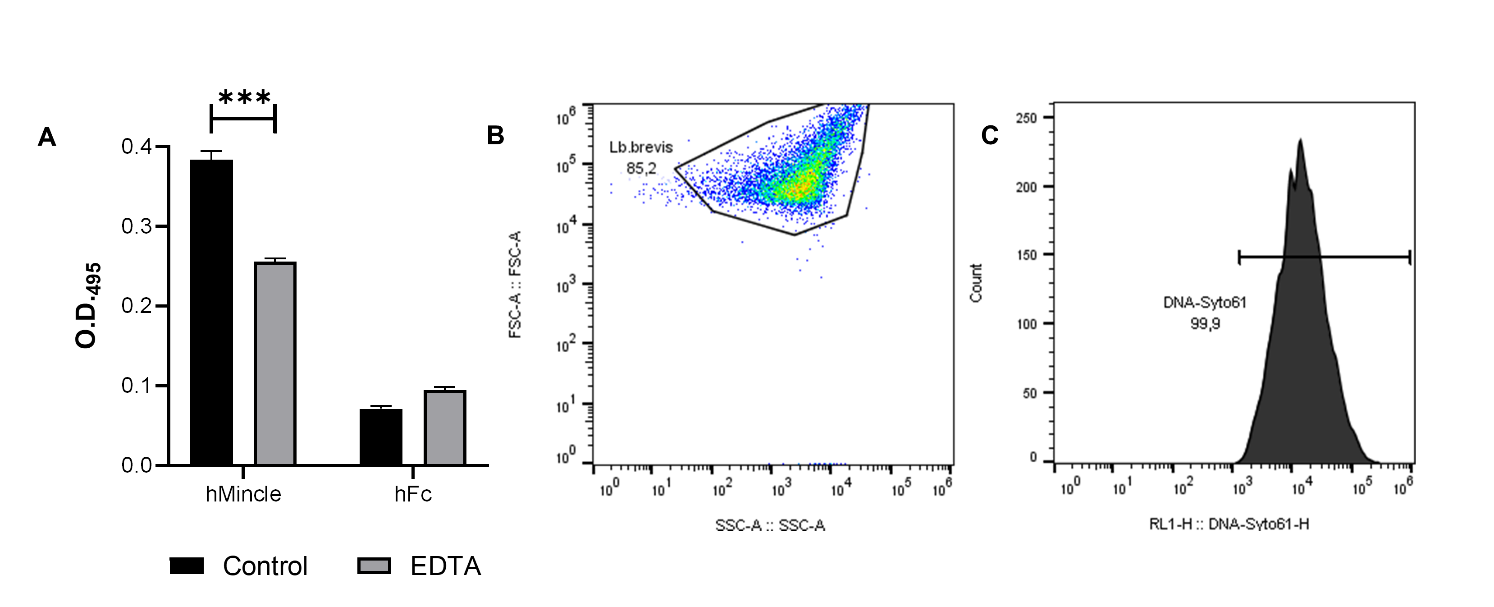


**Supplementary Figure 3.** Binding of human Mincle to S-layer and gating strategy**.** (A) Binding of commercial human-Mincle fusion protein to S-layer of *L.brevis* . Binding was measure at O.D 495. (B) Histoplots show the gating strategy to detect the binding of Mincle–hFc fusion proteins to S-layer in a representative experiment. Cells were discriminated from debris by gating on forward scatter (FSC) and side scatter (SSC). *L.brevis* was further gated on the DNA stain, which corresponds to SYTO61 positivity (C). Finally, SYTO61^+^ bacteria were analyzed for Mincle–hFc binding.


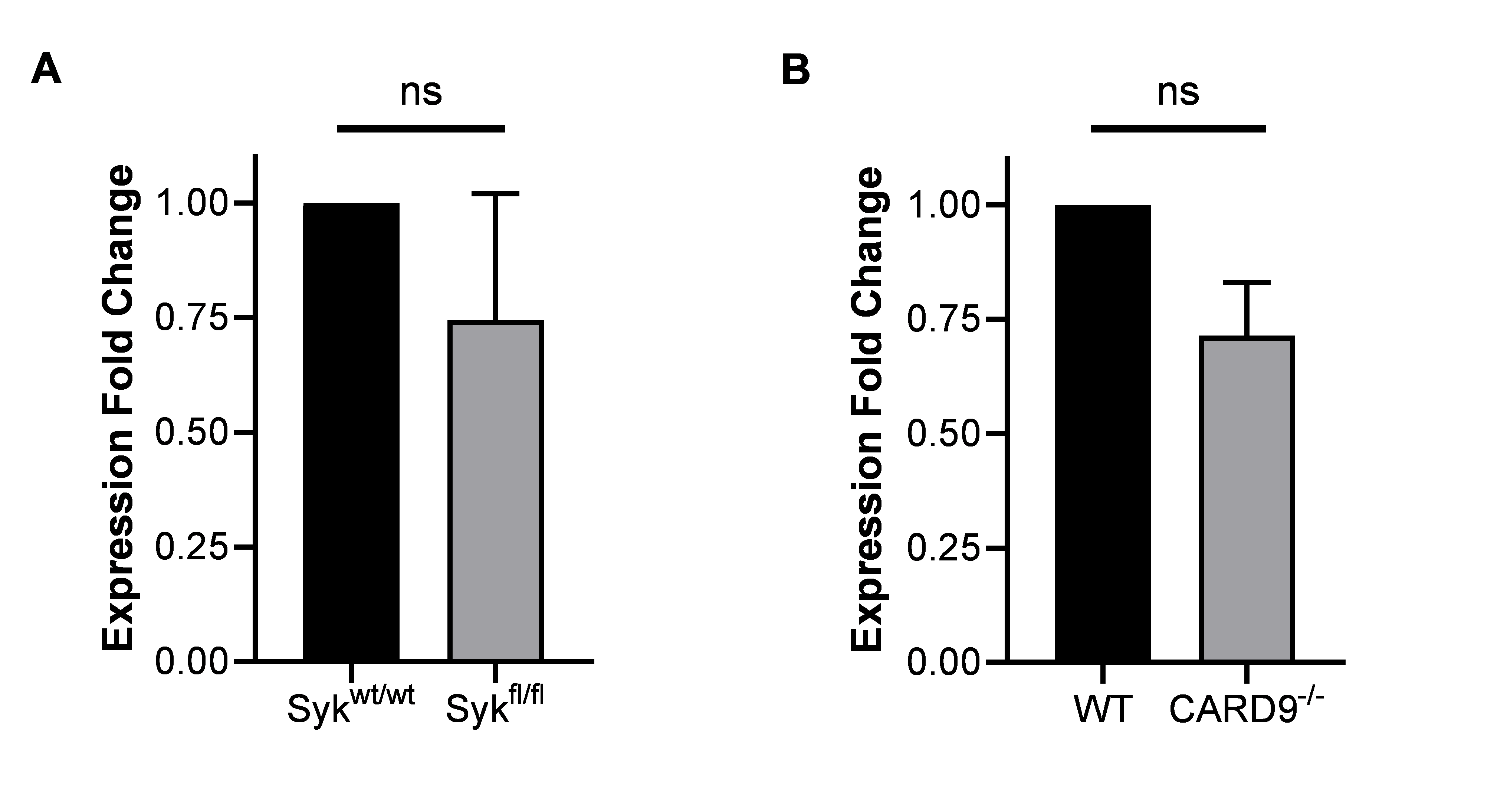


**Supplementary Figure 4.** qRT-PCR for Mincle expression in BMDCs from knock-out mice. (A) Relative expression of Mincle in CARD9^-/-^ BMDCs. (B) Relative expression of Mincle in Syk ^-/-^ BMDCs.
